# Iatrogenic Middle Cerebral Artery Ruptured Pseudoaneurysm Successfully Treated With a Pipeline Embolization Device

**DOI:** 10.31486/toj.19.0122

**Published:** 2021

**Authors:** Tyler Scullen, Mansour Mathkour, Jessica R. Carr, Aaron S. Dumont, Peter S. Amenta

**Affiliations:** ^1^ Department of Neurosurgery, Tulane Medical Center, New Orleans, LA; ^2^ Department of Neurosurgery, Ochsner Clinic Foundation, New Orleans, LA; ^3^ Tulane University School of Medicine, New Orleans, LA

**Keywords:** *Dual anti-platelet therapy*, *embolization device*, *intracranial aneurysm*, *middle cerebral artery*, *rupture*, *subarachnoid hemorrhage*

## Abstract

**Background:** Endovascular advances have shifted the treatment algorithms for traumatic intracranial pseudoaneurysms (IPs) from vessel sacrifice to reconstruction. The Pipeline embolization device (PED) is a flow-diverting stent that promotes endothelialization across the lesion and reconstitutes the parent vessel lumen.

**Case Report:** A 66-year-old male with a history of a right orbital apex lesion presented for biopsy with ophthalmology. Ophthalmology performed a right lateral orbitotomy complicated by brisk arterial bleeding from a proximal right middle cerebral artery (MCA) pseudoaneurysm. The MCA pseudoaneurysm was treated endovascularly with a PED, resulting in immediate stasis of contrast within the lesion without compilation. Interval follow-up angiograms 6 weeks and 6 months after the procedure showed no evidence of recurrence and a widely patent stent.

**Conclusion:** The PED provided a rapid, minimally invasive, and durable treatment option for an acutely ruptured IP. We illustrate that endovascular management with flow diversion can be effectively used in select cases and provides a way to reconstruct the damaged vessel lumen and obliterate the aneurysm.

## INTRODUCTION

Traumatic intracranial pseudoaneurysms (IPs) are rare lesions that usually occur as a result of penetrating head trauma or iatrogenic injury from surgical intervention.^1^ Compared to the more common saccular aneurysms, IPs lack a true wall and are contained by a thin layer of friable intima.^1^ As a result, clip ligation of these aneurysms is generally not used because of the high risk of intraoperative rupture and the lack of tissue for closing the clip.^1^ Instead, open intervention consists primarily of parent vessel sacrifice with or without bypass.^2^ Without treatment, IPs have a high rate of rupture and re-rupture, with associated significant morbidity and mortality.^3^

Endovascular advances have led to minimally invasive alternatives for management of traumatic IPs and shifted the treatment algorithm from vessel sacrifice to vessel reconstruction.^4^ Flow-diverting stents were initially introduced for the treatment of wide-neck aneurysms that were not routinely treated with traditional microsurgical and endovascular techniques.^4-7^ The Pipeline embolization device (PED) is a flow-diverting stent composed of interwoven cobalt-chromium and platinum-tungsten alloys that provides 30% to 35% coverage within the parent vessel.^1^ The high metal coverage diverts flow from the aneurysmal sac, promotes endothelialization across the neck, and reconstitutes the parent vessel lumen.^1^ The requirement of dual antiplatelet therapy (DAPT), however, limits the utility of PED use in the setting of acute subarachnoid hemorrhage (SAH).^4,6,7^ Despite this limitation, these stents are being applied to the management of clinical scenarios and pathologies beyond their on-label indications with increasing frequency.^4-7^

We describe the successful treatment of a ruptured middle cerebral artery (MCA) first (M1) segment iatrogenic IP in the acute setting with a PED.

## CASE REPORT

**Figure 1.**
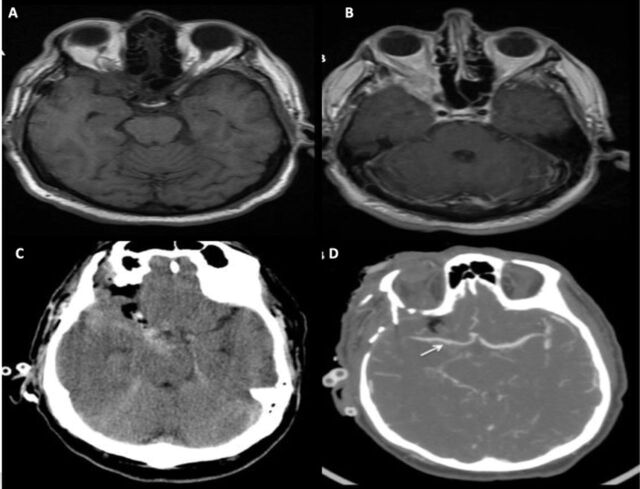
Axial magnetic resonance images of the brain (A) without and (B) with contrast show persistent enhancing right orbital apex mass prior to the planned ophthalmology procedure. (C) Axial computed tomography (CT) scan of the head demonstrates subarachnoid hemorrhage immediately following the transorbital approach to the orbital apex. (D) Axial CT angiography suggests mild irregularity of the right M1 segment (arrow).

A 66-year-old male with a history of right orbital apex lesion (Figures 1A and 1B) presented for biopsy with ophthalmology. The patient had undergone 2 nondiagnostic biopsies via pterional craniotomies at an outside institution, and the lesion had then been treated with 2 courses of external beam radiation (XRT). Outside records were unavailable, and the rationale behind the prior treatment without a diagnosis was unknown.

Ophthalmology performed a right lateral orbitotomy, and brisk arterial bleeding occurred during the biopsy. The hemorrhage was controlled with packing, and the procedure was aborted. When the patient awoke, he had transient left hemiplegia and dysarthria that promptly resolved. Neurosurgery was consulted after computed tomography demonstrated acute SAH in the basal cisterns and proximal right Sylvian fissure (Figures 1C and 1D).

**Figure 2.**
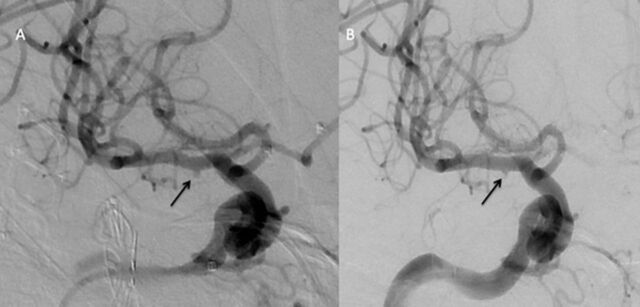
(A) Initial anterior-posterior digital subtraction angiography of the right internal carotid artery demonstrates a right M1 segment pseudoaneurysm (arrow) arising from the ventral surface of the proximal M1 segment. (B) Repeat injection immediately after Pipeline embolization device deployment shows stasis on contrast (arrow) in the late arterial phase.

Emergent cerebral digital subtraction angiography (DSA) demonstrated a 2 × 2-mm IP arising from the ventral surface of the proximal right M1 segment (Figure 2A). Considering the previous right-sided surgery and multiple rounds of XRT, microsurgical intervention was deemed a poor treatment option because of a heavily scarred operative field, fragility of the target lesion, and the emergent nature of the procedure. The patient received a loading dose via nasogastric tube of 600 mg of clopidogrel and 650 mg of aspirin in the angiography suite. A 3.25 × 14-mm PED was deployed within the right M1 segment across the IP, with immediate stasis of contrast within the lesion (Figure 2B).

**Figure 3.**
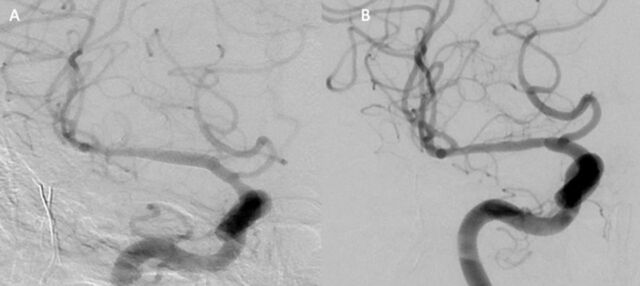
(A) Postoperative day 6 digital cerebral angiography (DSA) of the right internal carotid artery shows complete obliteration of the pseudoaneurysm. (B) Six-month follow-up DSA demonstrates persistent and complete obliteration and a widely patent Pipeline embolization device.

The patient awoke neurologically intact, and his platelet adenosine receptor P2Y12 response unit assay value (120 U) in the immediate postoperative period confirmed adequate platelet inhibition. The patient was continued on a maintenance dose of DAPT: daily 81 mg aspirin and 75 mg clopidogrel. DSA on postoperative day 6 demonstrated complete obliteration of the IP and restoration of the normal caliber and contour of the M1 segment (Figure 3A). The patient had an uncomplicated postoperative course and was discharged to an inpatient rehabilitation facility on the DAPT regimen for 6 months. Interval DSA 6 weeks and 6 months after the procedure showed no evidence of recurrence and a widely patent stent (Figure 3B). Clopidogrel was discontinued at 6 months, and aspirin 81 mg daily was continued for life.

## DISCUSSION

Hemorrhagic traumatic IPs are potentially fatal and associated with high morbidity and mortality.^8^ The significant risk of re-rupture in the acute setting warrants urgent intervention and elimination of the lesion.^1,8^ These lesions are rare, with the series largely limited to fewer than 10 patients.^4-7^ When IP is reported in the setting of iatrogenic^4-7,9^ trauma, basilar artery apex injury secondary to endoscopic third ventriculostomy^4,6^ and internal carotid artery injury during transsphenoidal approaches to the sella turcica are most commonly described.^1^

Our patient had significant arterial bleeding through the orbit and into the subarachnoid space immediately upon biopsy. The mechanism underlying the transient postoperative left-sided weakness and dysarthria was assumed to be vasospasm in the M1 segment following puncture of the vessel.

Endovascular intervention, particularly with flow diverters, has altered the management algorithm from vessel sacrifice with or without bypass to vessel reconstruction.^2,4-7^ The use of the PED in the setting of acute hemorrhage is off-label and remains controversial because of the requirement for extended DAPT regimens.^4-7^ In our case, microsurgical exclusion of the IP and bypass were ruled out because of the extensive postsurgical and postradiation changes in the operative field. Endovascular coiling of these lesions is associated with a high failure rate attributable to intraoperative rupture and the propensity of the lesions to enlarge and re-canalize postcoiling.^7^ Embolization of the parent vessel with liquid embolics would have assuredly resulted in a complete and disabling MCA territory infarct.^1^ The PED provided a minimally invasive technique for reconstructing the vessel lumen and obliterating the lesion.

The primary argument against the use of flow diversion in the setting of acute hemorrhage is the need for prolonged DAPT.^4^ These patients often require additional surgical procedures, such as shunting for cerebrospinal fluid diversion and decompressive craniectomies for elevated intracranial pressure. These procedures can be complicated or even prohibited by significant platelet inhibition.^4,6,7,10^ DAPT can be safely used despite the presence of hemorrhage.^2^ We argue that flow diversion for these traditionally challenging and aggressive lesions represents a viable treatment option in carefully selected patients with an acceptable risk-benefit profile,^4,6,7,10^ and this report adds to the existing literature supporting the use of flow diversion therapy in the setting of acutely ruptured iatrogenic IPs.^6,7^

## CONCLUSION

We report successful endovascular obliteration of an acutely ruptured iatrogenic IP using a PED, a rapid, minimally invasive, and durable treatment option. Ruptured IPs secondary to iatrogenic injury are rare but aggressive lesions that are prone to early re-rupture and are associated with high morbidity and mortality, necessitating prompt diagnosis and treatment. We illustrate that endovascular management with flow diversion can be effectively employed in select cases and used to reconstruct the damaged vessel lumen and obliterate the IP. The need for DAPT in the setting of acute hemorrhage may theoretically complicate the postoperative course.

## Notes

*This article meets the Accreditation Council for Graduate Medical Education and the American Board of Medical Specialties Maintenance of Certification competencies for Patient Care and Medical Knowledge.*
